# Lactylation in glioblastoma: mechanisms and therapeutic perspectives

**DOI:** 10.3389/fcell.2026.1940871

**Published:** 2026-10-20

**Authors:** Long Huang, Boyu Huang, Shizhen Kuang, Yaohua Yan

**Affiliations:** Department of Neurosurgery, Affiliated Hospital of Nantong University, Nantong, China

**Keywords:** epigenetics, glioblastoma, lactylation, metabolic reprogramming, therapeutic resistance

## Abstract

Glioblastoma (GBM) is one of the most aggressive primary malignant tumors in the central nervous system. It is characterized by significant metabolic reprogramming, obvious glycolysis dependence, and excessive lactate accumulation. Rather than being merely a metabolic by-product, lactate is increasingly recognized as a signaling metabolite that can reshape tumor biology. The discovery of lactylation as a post-translational modification has established a direct mechanistic link between lactate metabolism, epigenetic regulation, and protein function. Evidence from GBM and broader glioma models indicates that lactylation regulates various carcinogenic processes, including tumor cell proliferation, maintenance of stemness, DNA damage repair, immune evasion, and resistance to chemotherapy and radiotherapy. Through these processes, lactylation couples metabolic states to transcriptional and signaling networks, thereby promoting malignant progression and treatment failure. This review summarizes current understanding of the molecular mechanisms regulating lactylation and systematically explores its role in the progression of GBM, treatment resistance, and remodeling of the tumor immune microenvironment. We also assess emerging strategies targeting lactate and the main obstacles in its clinical translation. Overall, lactylation may serve as an important regulatory interface connecting metabolic reprogramming and the malignant phenotype of GBM; however, its value as a clinical biomarker or therapeutic target still requires further validation.

## 1 Introduction

Glioblastoma (GBM) is the most common and aggressive primary malignant tumor in the central nervous system, characterized by diffuse infiltration, rapid progression, and high resistance to conventional treatments (Price et al., 2024). Despite advances in neurosurgical techniques, maximal safe resection followed by concurrent radiotherapy and temozolomide (TMZ) and subsequent adjuvant TMZ remains the standard treatment for newly diagnosed GBM (Stupp et al., 2005; Weller et al., 2021). However, patients with MGMT promoter-methylated tumors generally derive greater benefit from TMZ, whereas the benefit is more limited in those with unmethylated tumors, particularly among elderly or frail patients (Hegi et al., 2005; Weller et al., 2021). Nevertheless, durable disease control remains difficult to achieve, and tumor recurrence is almost inevitable. Therefore, the median survival of GBM patients remains unsatisfactory. These clinical realities highlight the urgent need to elucidate the fundamental biological mechanisms driving the progression of GBM and treatment resistance.

Metabolic reprogramming is a prominent and therapeutically relevant feature of GBM. Its metabolic plasticity allows tumor cells to withstand hypoxia and nutrient stress within the tumor microenvironment (TME) (Flint et al., 2017). A key part of this adaptation is aerobic glycolysis, known as the Warburg effect, in which GBM cells favor glycolysis even when oxygen is available. Although this pathway yields less ATP, it supplies intermediates for rapid proliferation and produces large amounts of lactate (Wolf et al., 2011). Lactate accumulation is particularly prominent in hypoxic, highly glycolytic regions of GBM. In solid tumors, lactate levels can reach approximately 40 μmol/g wet tissue, roughly equivalent to 40 mM when expressed on a tissue-volume basis (Walenta and Mueller-Klieser, 2004). Lactate is not simply metabolic waste. It promotes angiogenesis, weakens antitumor immunity, and alters intracellular signaling, thereby reshaping the TME (Qian et al., 2023). This understanding has established lactate as an important signaling metabolite in cancer. However, early research focused mainly on its extracellular effects, leaving its direct role in post-translational regulation largely overlooked.

This view changed fundamentally in 2019, when Zhang et al. identified lysine lactylation as a new form of protein post-translational modification (Zhang et al., 2019). In the canonical enzymatic pathway, lactyl-CoA serves as the activated donor for the transfer of lactyl groups to lysine residues, thereby linking cellular metabolism directly to epigenetic regulation and protein function (Zhang et al., 2019; Liu et al., 2025). The discovery helped explain how lactate influences chromatin remodeling and gene transcription, revealing a previously unknown connection between metabolism and epigenetics. Since then, lactylation has attracted growing interest in several diseases, especially cancer (Li et al., 2022; Lv et al., 2025). Current evidence suggests that it promotes tumor progression through both histone and non-histone targets. Histone lactylation can reshape transcriptional programs involved in proliferation, stemness, and immune regulation. By contrast, non-histone lactylation may affect protein stability, enzyme activity, and signal transduction. For example, p53 lactylation weakens its DNA-binding capacity and suppresses pro-apoptotic transcription (Zong et al., 2024). Lactylation can also stabilize programmed death-ligand 1 (PD-L1), thereby promoting immune evasion and resistance to immunotherapy (Tong et al., 2024). Together, these findings position lactylation as a key link between metabolic signals, oncogenic pathways, and immune regulation.

Although lactylation is increasingly recognized as important in GBM, its mechanistic role remains incompletely understood. In particular, no systematic framework has yet connected lactylation-driven epigenetic reprogramming with tumor stemness, therapeutic resistance, and immune suppression. This review examines current advances in the regulation and biological functions of lactylation, with an emphasis on GBM. We further discuss its contribution to tumor progression, treatment resistance, and immune evasion. By bringing these findings together, we aim to provide a clearer framework for future mechanistic studies and translational research.

### 1.1 Literature search and evidence inclusion criteria

We searched PubMed, Web of Science Core Collection, and Scopus from inception to August 12, 2026. Search terms included “glioblastoma/GBM/glioma”, “lactylation/lysine lactylation/histone lactylation/non-histone lactylation”, “lactate metabolism”, and “glycolysis”, combined with AND/OR, and we manually checked references of included studies and reviews.

For inclusion, we prioritized English, peer-reviewed original studies; reviews were used only for background information and reference tracking. Evidence was classified as direct GBM evidence, broader glioma evidence, or evidence from other diseases. Retracted articles were excluded. When duplicate reports were identified, the most complete version was retained. Preprints were not used as core evidence. Bioinformatics analyses based solely on lactylation-related gene expression were treated as suggestive, whereas lactate metabolism studies without direct lactylation detection were not considered direct evidence of the lactylation mechanism.

## 2 Shared biological roles of lactylation across cancers

Recent studies have increasingly focused on how lactylation influences tumor biology. As a protein post-translational modification, lactylation can regulate gene expression through histone modification and modulate protein function through non-histone modification, thereby contributing to cancer initiation, progression, dissemination, and therapeutic resistance (He et al., 2024; Rho and Hay, 2025; Sui et al., 2025). This section outlines the general biological roles of lactylation across cancer types and provides a foundation for the subsequent discussion of its specific mechanisms and functional consequences in GBM.

### 2.1 Lactylation and cancer metabolic reprogramming

A hallmark of tumor metabolic reprogramming is the preference for glycolysis even when oxygen is available, resulting in excessive lactate accumulation (Brown and Ganapathy, 2020). Rather than remaining a metabolic by-product, lactate can drive the covalent modification of histone and non-histone proteins, helping cancer cells adapt their metabolism. In cervical cancer, DCBLD1 lactylation inhibits the autophagic degradation of G6PD. This activates the pentose phosphate pathway and supports tumor proliferation (Meng et al., 2024). Lactylation can also alter metabolism by impairing enzyme function. In hepatocellular carcinoma, AK2 lactylation at K28 reduces its enzymatic activity and accelerates tumor progression (Yang et al., 2023). Together, these mechanisms can create a positive feedback loop: glycolysis increases lactate production, lactate drives lactylation, and lactylation further reinforces metabolic adaptation.

### 2.2 Lactylation, tumor stemness, and malignant progression

Cancer stem cells can self-renew and differentiate, making them important drivers of tumor recurrence and metastasis (Batlle and Clevers, 2017). Evidence from several cancers suggests that lactylation helps preserve these stem-like properties. In hypoxic colon cancer cells, elevated histone H3 lysine 18 lactylation (H3K18la) at the promoters of stemness-related genes enhances their transcription, supporting stemness and tumorsphere formation (Zhang Q. et al., 2025). In esophageal cancer, SHMT2 lactylation increases *MTHFD1L* expression and glycolysis, which strengthens tumor stemness and malignant behavior (Qiao et al., 2024). Lactylation also contributes to epithelial–mesenchymal transition (EMT), a process closely linked to metastatic spread (Nieto et al., 2016). In pancreatic cancer, Snail1 lactylation reinforces EMT and enhances cell migration and invasion (Zhao et al., 2024). In colorectal cancer, *KRAS* mutation increases histone H3 lysine 9 lactylation (H3K9la), driving progression and metastasis through the upregulation of the cholesterol transporter *GRAMD1A* (Zhang et al., 2025b). Overall, lactylation links metabolic changes to epigenetic and protein regulation, helping maintain cancer stemness and promote malignant progression across tumor types.

### 2.3 Lactylation and cell death

Tumor cells often develop resistance to programmed cell death. Lactylation can support their survival by regulating autophagy and apoptosis. For example, VPS34 lactylation promotes autophagosome formation, helping tumor cells maintain metabolic balance and survive nutrient stress (Jia et al., 2023). In uveal melanoma, H3K18la increases expression of the N^6^-methyladenosine (m^6^A) reader YTHDF2. This accelerates the degradation of tumor-suppressive mRNAs, including *PER1* and *TP53*, suppressing apoptosis and promoting tumor progression (Yu et al., 2021). Lactylation also affects ferroptosis by strengthening antioxidant defenses, reshaping lipid metabolism, or changing the stability of ferroptosis-related proteins. In lung adenocarcinoma, ferroptosis-induced SUMO2 lactylation promotes ACSL4 degradation, creating a negative feedback loop that limits ferroptosis (Shan et al., 2025). Its role in cuproptosis is also gaining attention. In gastric cancer, copper stress induces METTL16-K229la, which enhances METTL16-mediated m^6^A modification and stabilizes *FDX1* mRNA, thereby increasing *FDX1* expression, promoting cuproptosis, and enhancing sensitivity to elesclomol (Sun L. et al., 2023).

### 2.4 Lactylation and immune evasion

Lactate accumulation in the TME plays an important role in immune evasion. Beyond its metabolic effects, lactate-driven lactylation can reprogram immune cells, weaken immune surveillance, and promote an immunosuppressive TME. In colorectal cancer, METTL1, PKM2, and H3K9la form a positive feedback loop that increases expression of the immune checkpoint molecule CD155, thereby suppressing natural killer cell and CD8^+^ T-cell activity (Wang F. et al., 2024). MOESIN lactylation also enhances TGF-β signaling and promotes regulatory T-cell generation, further limiting antitumor immunity (Gu et al., 2022). Together, these findings identify lactylation as an important regulator of tumor immune suppression and suggest that targeting this modification may improve responses to current immunotherapies.

### 2.5 Lactylation and treatment resistance

Therapeutic resistance remains a major challenge in clinical oncology, and lactylation can contribute through several mechanisms. In bladder cancer, H3K18la induces cisplatin resistance by increasing *YY1* and *YBX1* expression (Li F. et al., 2024). In colorectal cancer, tumor-derived lactate promotes H3K18la-dependent expression of the autophagy enhancer *RUBCNL*, reducing sensitivity to bevacizumab (Li W. et al., 2024). In pancreatic ductal adenocarcinoma, ENSA-K63la activates SRC–STAT3–CCL2 signaling, recruits macrophages, and weakens cytotoxic T-cell activity, ultimately driving immunotherapy resistance (Sun K. et al., 2025). These findings suggest that targeting lactylation may help reverse different forms of treatment resistance, particularly in combination therapies. More broadly, lactylation contributes to metabolic reprogramming, stemness, immune evasion, invasion, metastasis, and therapeutic resistance across cancers. In GBM, high glycolytic activity, hypoxia, and lactate accumulation provide conditions that favor lactylation, making this modification especially relevant to disease progression. However, mechanisms identified in other cancers cannot be assumed to operate in GBM. They should instead guide the design of GBM-specific mechanistic studies.

## 3 Lactylation in GBM

Building on this disease-scope classification, mechanistic studies can be grouped into four evidence categories: (1) site-specific lactylation established by residue identification together with mutagenesis, competitive peptides, or functional rescue; (2) histone lactylation defined by a specific site, chromatin enrichment, and a related transcriptional change; (3) effects of lactate exposure or metabolic intervention without proof that lactylation directly mediates the response; and (4) bioinformatic subtyping or prognostic models derived from lactylation-related gene sets (Table 1).

**TABLE 1:** Mechanistic evidence for histone and non-histone lactylation in glioblastoma and broader glioma studies.

Lactylation event/site	Upstream signal	Downstream target/pathway	Principal effect	Experimental system and scope	Key evidence	Evidence class and reference
Histone lactylation regulating gene transcription						
H3K18la	Hypoxia–HIF-1α → glycolysis and lactate accumulation	H3K18la-associated *YTHDF2* upregulation → BNIP3-dependent mitophagy	Promotes proliferation, migration, invasion, and hypoxic adaptation	Glioma cells and xenografts; broader glioma evidence	ChIP, YTHDF2/BNIP3 perturbation, in vivo validation	II; (Dong et al., 2025a)
H3K18la	Nuclear GTPSCS converts lactate and CoA to lactyl-CoA and cooperates with p300	H3K18la enrichment at the *GDF15* promoter → increased *GDF15* transcription	Promotes proliferation, tumorigenicity, and radioresistance	Glioma cells, PDXs, and mixed-grade glioma tissues	LC-MS, enzymology, ChIP-seq, PDX validation	II; (Liu et al., 2025)
Macrophage H3K18la	Hypoxic glioma-derived lactate → uptake by macrophage MCT1	H3K18la enrichment at the macrophage *TNFSF9* promoter → increased *TNFSF9* transcription	Promotes M2-like polarization and glioma progression	U87/U251-macrophage coculture and xenografts; broader glioma evidence	ChIP-seq, MCT1 perturbation, polarization and in vivo assays	II; (Li et al., 2024c)
H4K16la	SUCLG2–DLAT interaction and altered lactate metabolism	H4K16la occupancy at *BEST1*, *GRAMD4*, and *MBD6* regulatory regions	Supports GBM-cell proliferation and limits apoptosis	U251, LN229, and xenografts; conventional cell-line evidence	SUCLG2 knockdown, DLAT interaction, CUT&Tag, ChIP-qPCR	II; (Li et al., 2025a)
H4K8la	Lactate accumulation	H4K8la enrichment at the *NUPR1* promoter → increased *NUPR1* transcription → autophagosome–lysosome fusion	Enhances protective autophagy and GBM progression	GBM cells, patient-derived cells/tissues, and in vivo models	CUT&Tag, ChIP-qPCR, autophagic flux, NUPR1 perturbation	II; (Zhao et al., 2026a)
H3K18la	GSC glycolysis/lactate increase	H3K18la enrichment at the *USP4* promoter → increased *USP4* transcription; USP4-mediated ANXA2 stabilization and Y24 phosphorylation → BMX–STAT3	Maintains GSCs and radioresistance	Patient-derived GSCs and xenografts	ChIP-qPCR, deubiquitination, stemness and radiotherapy assays	II; (Tu et al., 2025)
H3K9la	TMZ resistance-associated lactate accumulation	H3K9la enrichment at the *LUC7L2* promoter → increased *LUC7L2* transcription → *MLH1* intron 7 retention and reduced MLH1 expression → impaired MMR	Promotes TMZ resistance	TMZ-resistant cells, primary/recurrent GBM tissues, and animal models	CUT&Tag, SLAM-seq, splicing and drug-sensitivity assays	II; (Yue et al., 2024)
H3K9la	Reduced nuclear SIRT6-mediated H3K9 delactylation	Increased H3K9la at the *MGMT* promoter → increased *MGMT* transcription	Enhances DNA repair and TMZ resistance	TMZ-resistant cells, GBM tissues, and animal models	ChIP-qPCR, SIRT6 mutants, drug-sensitivity assays	II; (Zou et al., 2025)
Histone H3 Kla (site unresolved)	Warburg effect and lactate accumulation; NF-κB activation	Increased *LINC01127* transcription → cis-regulation of *MAP4K4* → JNK/NF-κB signaling	Promotes GSC self-renewal and tumorigenicity	Patient-derived GSCs and xenografts	Pan-H3Kla, promoter ChIP, pathway perturbation	III; (Li et al., 2023)
Pan-histone Kla (site unresolved)	Microenvironmental lactate; CBX3 biases p300 toward lactyl-CoA-dependent histone lactylation	Site-unresolved histone Kla at immune-regulatory promoters, including *CD47* → immunosuppressive transcriptional programs	Suppresses microglial/macrophage phagocytosis and promotes immune escape	Patient-derived GSCs, myeloid coculture, and xenografts	Co-IP, enzymology, ChIP, phagocytosis and in vivo assays	III; (Wang et al., 2024b)
MDM histone Kla (site unresolved)	GBM-derived factors → PERK–ATF4–GLUT1 → MDM glycolysis and intracellular lactate	Site-unresolved histone Kla at the *IL10* promoter → increased IL-10 expression	Establishes immunosuppressive MDMs	Human GBM samples, BMDMs, and immunocompetent models	^13^C tracing, CUT&RUN-qPCR, myeloid PERK deletion	III; (De Leo et al., 2024)
H3K18la	Lactate accumulation	H3K18la enrichment at the *BMAL1* promoter → increased *BMAL1* transcription	Contributes to VEGFA activation and bevacizumab resistance	GBM cells, PDXs, animal models, and five clinical cohorts	ChIP-qPCR, lactate/MCT perturbation, treatment validation	II; (Wang et al., 2026)
Site-specific non-histone lactylation						
PTBP1-K436la	GSC glycolysis/lactate increase; SIRT1 delactylation	Reduced TRIM21 binding and proteasomal degradation of PTBP1; increased PTBP1 RNA binding → stabilization of *PFKFB4* mRNA	Maintains GSCs and a glycolytic positive-feedback loop	GSCs and animal models	LC-MS/MS, K436R, RIP, in vivo validation	I; (Zhou et al., 2025)
XRCC1-K247la	ALDH1A3–PKM2 interaction → PKM2 tetramerization and lactate accumulation	Increased XRCC1–importin-α binding and nuclear translocation → enhanced SSB repair	Promotes radioresistance and TMZ resistance	Patient-derived GSCs, PDOs, xenografts, and GBM samples	LC-MS/MS, site-specific antibody, K247R, comet assay	I; (Li et al., 2024b)
RPA1-K88la	Lactate → KAT2B-mediated H4K12la at the *HAT1* promoter → increased *HAT1* transcription; HAT1-K15la enhances HAT1 activity	HAT1-catalyzed RPA1-K88la → increased RPA1–ssDNA binding → HR	Promotes DSB repair and radioresistance	LN229, LN18, and intracranial xenografts; limited clinical validation	MS, mutagenesis, in vitro lactylation, HR assay, competitive peptide	I; (Zhao et al., 2026b)
HDAC1-K200la	MES-MDM-derived TNF → CELSR2/p65 signaling in GBM cells → HDAC1-K200la	HDAC1-K200la → EPAS1/CEBPD-dependent MES-like transcriptional program	Promotes MES-like transition and TMZ resistance	Patient samples, PDOs, coculture, and orthotopic models	Single-cell/spatial transcriptomics, MS, K200R, drug-sensitivity assays	I; (Gao et al., 2026)
KU70-K317la	TAM MCT4-mediated lactate export → GSC MCT1-mediated uptake	KU70-K317la → increased NHEJ → reduced cytosolic DNA → suppressed cGAS–STING signaling	Enhances DNA repair, maintains GSC stemness, and promotes immune suppression	GSC-TAM coculture and immunocompetent intracranial models	MS, K317 mutation, NHEJ, immune and anti-PD-1 assays	I; (Li et al., 2026)
BMAL1-K123la	Lactate accumulation	BMAL1-K123la → increased TUBA1C binding → BMAL1 nuclear translocation → increased VEGFA transcription	Promotes bevacizumab resistance	GBM cells, PDXs, animal models, and five clinical cohorts	IP-MS, Co-IP, K123R, treatment validation	I; (Wang et al., 2026)

The evidence classes were developed for this review and are not GRADE categories. Class I: a specific non-histone lactylation site identified and functionally validated by site mutation, a site-specific assay, a competitive peptide, or rescue. Class II: a mechanistic link supported by a defined histone lactylation site, chromatin enrichment, and functional perturbation, but without causal validation by mutation of the individual histone residue. Class III: a biological effect linked to manipulation of lactate metabolism or global/site-unresolved Kla without functional validation of a defined lactylated residue. Conventional GBM-derived cell lines or broader glioma models are not equivalent to validation in patient-derived GBM defined by current molecular diagnostic criteria.

Abbreviations: BMDM, bone-marrow-derived macrophage; ChIP, chromatin immunoprecipitation; Co-IP, co-immunoprecipitation; CUT&RUN, cleavage under targets and release using nuclease; CUT&Tag, cleavage under targets and tagmentation; DSB, double-strand break; GBM, glioblastoma; GSC, glioma stem cell; HIF-1α, hypoxia-inducible factor 1α; HR, homologous recombination; Kla, lysine lactylation; LC-MS/MS, liquid chromatography–tandem mass spectrometry; MCT, monocarboxylate transporter; MDM, monocyte-derived macrophage; MES, mesenchymal-like; MMR, mismatch repair; MS, mass spectrometry; NHEJ, non-homologous end joining; PDO, patient-derived organoid; PDX, patient-derived xenograft; qPCR, quantitative polymerase chain reaction; RIP, RNA immunoprecipitation; ssDNA, single-stranded DNA; SSB, single-strand break; TAM, tumor-associated macrophage; TMZ, temozolomide.

### 3.1 Lactate accumulation and the regulation of lactylation in GBM

Regional metabolic heterogeneity is a characteristic feature of GBM (Miranda-Gonçalves et al., 2016; Guyon et al., 2022). Tumor cells located in relatively well-oxygenated areas may maintain active glycolysis and release considerable amounts of lactate. In oxygen-deficient regions, by contrast, hypoxia-inducible factor (HIF)-dependent metabolic responses further favor lactate production and accumulation (Miranda-Gonçalves et al., 2016; Guyon et al., 2022). Inefficient removal of lactate may additionally contribute to its local enrichment (Torrini et al., 2022). Lactate is therefore more than a passive end product of glucose metabolism. It can move between tumor regions and cell populations, supporting metabolic communication within the TME (Guyon et al., 2022; Wang S. et al., 2024). It also supplies the metabolic substrate required for the lactylation of histone and non-histone proteins (Zhang et al., 2019; Wang S. et al., 2024; Mao et al., 2024). Understanding how lactate is produced, transported, and used within cells is therefore essential for defining how lactylation arises and is regulated in GBM.

#### 3.1.1 Hypoxia and enhanced glycolysis drive lactate accumulation

GBM cells often favor aerobic glycolysis, commonly known as the Warburg effect (Wolf et al., 2011; Flavahan et al., 2013; Stanke et al., 2021). Even when oxygen is available, some tumor cells convert much of their glucose-derived pyruvate into lactate rather than fully oxidizing it in mitochondria. Although glycolysis produces less ATP per glucose molecule, it generates energy rapidly and supplies intermediates needed for tumor cell proliferation. Increased expression or activity of GLUT1, GLUT3, HK2, PFK, PKM2, and LDHA further enhances glucose uptake and glycolytic flux, promoting lactate production (Wolf et al., 2011; Flavahan et al., 2013; Stanke et al., 2021). Importantly, mitochondrial glucose oxidation is not lost. Glycolysis and oxidative metabolism can coexist within the same tumor, reflecting the metabolic plasticity and heterogeneity of GBM (Marin-Valencia et al., 2012; Stanke et al., 2021). Hypoxia shifts this balance further toward lactate production (Brat et al., 2004; Miranda-Gonçalves et al., 2016). Rapid tumor growth and poorly organized blood vessels limit oxygen delivery, giving rise to extensive hypoxic regions in GBM (Brat et al., 2004). In response, HIF-1α increases glucose uptake and induces factors involved in glycolysis, including lactate dehydrogenase A (LDHA) and pyruvate dehydrogenase kinase 1 (PDK1). These changes divert pyruvate away from the tricarboxylic acid cycle and favor its conversion to lactate (Kim et al., 2006; Miranda-Gonçalves et al., 2016; Stanke et al., 2021). Hypoxic signaling therefore strengthens glycolytic metabolism and contributes to lactate accumulation in GBM.

#### 3.1.2 Lactate transport, metabolic symbiosis, and nuclear utilization

Lactate transport across GBM cell membranes is mediated mainly by monocarboxylate transporters (MCTs), particularly MCT1 and MCT4. MCT4 is often enriched in hypoxic, highly glycolytic cells and generally exports lactate together with protons. By contrast, MCT1 commonly supports lactate uptake in better-oxygenated tumor cells. These roles are not fixed, however, because the direction of transport depends on the lactate and proton gradients across the plasma membrane (Halestrap, 2012). This transport system supports a spatial division of metabolism within GBM. Hypoxic cells produce and release lactate, whereas more oxygenated cells can take it up, convert it to pyruvate, and use it as fuel. Such metabolic cooperation, often termed metabolic symbiosis, reduces competition for glucose among tumor cell populations (Sonveaux et al., 2008). Lactate can also move between malignant and nonmalignant cells in the surrounding microenvironment, serving as an intermediary in metabolic communication (Longhitano et al., 2022). Once inside the cell, lactate may support both energy metabolism and the generation of substrates required for nuclear protein lactylation (Zhang et al., 2019). How lactate enters the nucleus remains unclear, as no nucleus-specific transporter has been identified. Because lactate is small and nuclear pores allow the passage of small metabolites, it may equilibrate between the cytoplasm and nucleus through these pores. This explanation remains plausible but has not yet been directly demonstrated.

#### 3.1.3 Lactyl donors and enzymatic regulation of lactylation

Protein lactylation involves the covalent addition of a lactyl group to a lysine residue. Before enzymatic incorporation into a protein substrate, the lactate-derived group generally needs to be metabolically activated. One proposed activated donor is L-lactyl-CoA, which can be generated from L-lactate. Because L-lactyl-CoA is present at very low concentrations in mammalian cells, its local synthesis and subcellular availability may be more important for lactylation activity than its overall cellular abundance (Zhang et al., 2019; Varner et al., 2020). At least two mechanisms have been proposed for enzymatic L-lactylation. In the lactyl-CoA-dependent mechanism, enzymes such as GTPSCS or ACSS2 have been implicated in lactyl-CoA production. Acyltransferases may then use this activated donor to transfer lactyl groups to lysine residues: p300 has been implicated in histone lactylation, whereas ACSS2 has been shown to cooperate with KAT2A as a histone lactyltransferase in tumor models (Zhang et al., 2019; Zhu et al., 2025; Rho and Hay, 2025; Liu et al., 2025). A second mechanism involves AARS1 and AARS2. These enzymes use ATP to activate lactate and catalyze protein lactylation without relying on lactyl-CoA as an intermediate (Zong et al., 2024; Rho and Hay, 2025). The biological relevance of these mechanisms may vary with the cellular context and protein substrate. In glioma, evidence for the lactyl-CoA-dependent route has been reported in association with the H3K18la–GDF15 regulatory axis. However, this finding does not establish that GTPSCS controls all forms of histone lactylation. The AARS1- and AARS2-mediated mechanism may help explain how widespread protein lactylation occurs despite the scarcity of lactyl-CoA. Nevertheless, current evidence for lactylation mediated by AARS1 and AARS2 comes mainly from non-histone substrates, and its contribution to GBM has yet to be directly established (Zong et al., 2024; Rho and Hay, 2025; Liu et al., 2025). Lysine lactylation can also occur through a chemically and biologically distinct route. During glycolysis, methylglyoxal is processed by the glyoxalase system to generate S-D-lactoylglutathione. Through a nonenzymatic reaction, this intermediate can donate a D-lactyl group to protein lysine residues, producing lysine D-lactylation. Accordingly, D-lactylation differs from histone L-lactylation in its metabolic origin, stereochemical configuration, and potential biological function (Gaffney et al., 2020). Lactylation is reversible rather than permanent. Class I histone deacetylases 1–3 (HDAC1–3), together with SIRT1, SIRT2, SIRT3, and SIRT6, have been reported to remove lactyl groups from modified proteins. The balance between lactyl-group deposition and removal therefore determines the abundance and duration of lactylation signals (Rho and Hay, 2025; Moreno-Yruela et al., 2022).

#### 3.1.4 The nonlinear relationship between glycolysis and lactylation

Hypoxia and exogenous lactate supplementation can increase protein lactylation, whereas inhibiting glycolysis or lactate production generally reduces it (Zhang et al., 2019; Liu et al., 2025). These findings support a functional link between glycolytic metabolism and lactylation. Still, the two do not always rise in parallel, and greater glycolytic flux does not necessarily lead to a matching increase in protein lactylation. This is because several regulatory steps lie between lactate production and protein modification. Intracellular lactate levels reflect the balance among production, uptake, export, and utilization. Thus, rapid glycolysis may occur without substantial lactate accumulation when MCT-mediated export is highly active (Halestrap, 2012). Lactylation also depends on the conversion of lactate into an activated donor and on the activities of writer and eraser enzymes (Zhang et al., 2019; Liu et al., 2025; Moreno-Yruela et al., 2022). Properties of the substrate itself, including protein conformation, lysine-site accessibility, and subcellular localization, further influence whether a protein is efficiently lactylated (Liu et al., 2025; Moreno-Yruela et al., 2022). Glycolytic activity therefore provides an important metabolic prerequisite for lactylation, but it does not by itself determine the overall lactylation landscape.

### 3.2 Functions and mechanisms of histone lactylation in GBM

Since histone lactylation was recognized as an important metabolic and epigenetic modification, growing attention has focused on how it promotes GBM progression by shaping chromatin accessibility and transcriptional programs (Liu et al., 2025). Recent studies suggest that lactate accumulation caused by hypoxia and increased glycolysis is a key upstream event that reprograms histone lactylation in GBM. This process contributes to tumor cell proliferation, invasion, and adaptive evolution (Dong F. et al., 2025; Li W. et al., 2025). Together, these findings position histone lactylation as a central epigenetic interface linking metabolic reprogramming to the evolution of malignant phenotypes in GBM.

#### 3.2.1 Promotion of GBM cell proliferation and survival

A recent study identified H4K8la enrichment at the *NUPR1* promoter, which increased *NUPR1* transcription, promoted autophagosome–lysosome fusion, and maintained protective autophagy. The H4K8la–NUPR1 axis was examined in GBM cells, patient tissues, and *in vivo* models. Supporting evidence included cleavage under targets and tagmentation (CUT&Tag), chromatin immunoprecipitation–quantitative PCR (ChIP–qPCR), autophagic flux assays, and functional perturbation of NUPR1. The study therefore provides site-resolved chromatin evidence together with downstream functional validation. However, it does not establish causality by mutating the histone residue itself (Zhao J. et al., 2026).

Multiple studies suggest that lactate-driven histone lactylation can directly reshape the epigenetic state of promoter regions and activate transcriptional programs involved in tumor cell proliferation and survival. In the hypoxic microenvironment, HIF-1α-induced H3K18la increases *YTHDF2* expression and enhances BNIP3-dependent mitophagy. Together, these changes increase lactate production and global histone lysine lactylation, maintain the transcription of proliferation- and invasion-related genes, and reinforce metabolic–epigenetic coupling in preclinical glioma models (Dong F. et al., 2025). The metabolic enzyme SUCLG2 has also emerged as a key regulator of histone lactylation in GBM. SUCLG2 knockdown was accompanied by reductions in lactate levels and H4K16 lactylation (H4K16la). Enrichment of H4K16la at the promoters of genes involved in proliferation and apoptosis enhances pro-proliferative gene expression while suppressing pro-apoptotic signaling. This shift promotes GBM cell proliferation and inhibits apoptosis. SUCLG2 knockdown also induces cell-cycle arrest and apoptosis and restricts tumor growth (Li W. et al., 2025). At the level of lactyl-CoA supply, nuclear GTPSCS catalyzes lactyl-CoA synthesis and interacts with p300 to increase H3K18la at the *GDF15* promoter. This enhances *GDF15* transcription and promotes glioma proliferation and radioresistance (Liu et al., 2025). Taken together, these studies show how this axis couples glycolytic reprogramming to the epigenetic activation of oncogenic transcriptional programs, thereby supporting GBM cell proliferation and survival.

#### 3.2.2 Maintenance of GBM stemness

Histone lactylation can help maintain the glioma stem cell (GSC) phenotype by activating both protein-coding genes and noncoding RNAs. Tu et al. showed that H3K18la upregulates *USP4* transcription, and the resulting increase in USP4 protein stabilizes ANXA2 through deubiquitination. This pathway maintains the GSC phenotype and promotes tumor progression (Tu et al., 2025). Histone lactylation also activates LINC01127 transcription. This long noncoding RNA regulates *MAP4K4* in cis and activates JNK signaling, thereby enhancing GSC self-renewal and supporting sustained tumor growth and malignant progression (Li et al., 2023). However, direct evidence linking noncoding RNA regulation by histone lactylation to GBM stemness remains limited. Future studies should investigate this mechanism and determine how it maintains stemness across different GBM cellular states. Overall, histone lactylation shapes the GBM phenotype through both classical protein-coding genes and noncoding RNA networks.

#### 3.2.3 Mediation of treatment resistance in GBM

Histone lactylation may also help GBM adapt to antiangiogenic therapy. A recent study showed that lactate increases H3K18la at the *ARNTL* promoter, thereby promoting *ARNTL* transcription and BMAL1 expression. At the same time, lactate induces BMAL1-K123la, strengthens the interaction between BMAL1 and TUBA1C, and promotes the nuclear translocation of BMAL1. These two regulatory mechanisms converge to increase *VEGFA* transcription and reduce sensitivity to bevacizumab. The BMAL1-K123R mutation, *ARNTL* silencing, and inhibition of lactate transport each partially restored treatment sensitivity. These effects were examined in GBM cells, patient-derived xenografts, additional animal models, and multiple clinical cohorts (Wang et al., 2026).

Lactate-driven histone lactylation also plays a critical role in GBM resistance to TMZ. Yue et al. found that lactate-induced H3K9la promotes *MLH1* intron retention through the splicing factor LUC7L2. This impairs DNA mismatch repair and markedly reduces the sensitivity of GBM cells to TMZ (Yue et al., 2024). Later studies showed that targeting this metabolic and epigenetic axis can reverse resistance. PPARα activation reduces lactate production and H3K18la, restoring the response to TMZ (Wang Z. C. et al., 2025). Beyond metabolic intervention, SIRT6 activation promotes H3K9 delactylation and reduces *MGMT* expression, which also restores TMZ sensitivity in GBM cells (Zou et al., 2025). Together, these findings define histone lactylation as one experimentally supported contributor to drug resistance in GBM and support the development of combination strategies targeting this axis.

#### 3.2.4 Promotion of immune evasion in GBM

At the level of tumor immune regulation, lactate-driven histone lactylation shapes an immunosuppressive TME through several mechanisms. Histone lactylation can cooperate with the heterochromatin protein CBX3 and increase the substrate specificity of p300 for lactyl-CoA. This activates transcriptional programs involving immunosuppressive genes such as *CD47*, thereby limiting microglial and macrophage phagocytosis of GBM cells. The resulting immune evasion contributes to disease progression (Wang S. et al., 2024). Histone lactylation induced by enhanced glucose metabolism also strengthens the immunosuppressive activity of monocyte-derived macrophages and weakens their antitumor function. These changes help establish an immunosuppressive TME and accelerate GBM progression (De Leo et al., 2024). Under hypoxic conditions, glioma-derived lactate is taken up by macrophages through MCT1, where it increases H3K18la-associated *TNFSF9* transcription and promotes M2-like polarization. This intercellular lactate-driven mechanism reinforces immunosuppression and promotes glioma progression (Li M. et al., 2024). Together, these findings show how lactylation supports immune evasion through multiple pathways and immune cell populations.

### 3.3 Functions and mechanisms of non-histone lactylation in GBM

Beyond histones, lactylation also affects a wide range of non-histone proteins, although its functional significance in GBM is only beginning to be understood. Recent studies suggest that this modification regulates signal transduction, protein stability, and immune responses (Xie et al., 2025). Compared with histone lactylation, it may allow GBM cells to respond more rapidly and flexibly to changes in their cellular context.

#### 3.3.1 Maintenance of GBM stemness and promotion of malignant progression

Notably, non-histone lactylation can directly reinforce the glycolytic phenotype that drives its formation, thereby maintaining GBM stemness and promoting malignant progression. In GSCs, lactylation of the RNA-binding protein PTBP1 at lysine 436 (PTBP1-K436la) stabilizes *PFKFB4* mRNA, increases PFKFB4 protein abundance, enhances glycolytic flux and lactate production, and sustains GSC self-renewal. This process establishes a positive feedback loop of lactate accumulation–PTBP1 lactylation–enhanced glycolysis that promotes GBM progression (Zhou et al., 2025). These findings identify non-histone lactylation as an important mechanism maintaining GBM stemness and malignant phenotypes; however, few relevant substrates have been defined, and additional mechanisms remain to be investigated. Another potentially relevant non-histone substrate is p53. Zong et al. showed that AARS1 senses intracellular lactate and catalyzes p53 lactylation at K120 and K139, thereby impairing p53 DNA binding, phase separation, and tumor-suppressive transcription (Zong et al., 2024). This mechanism may be relevant to GBM, in which TP53 mutations and other alterations of the p53 pathway are frequently observed; however, whether different TP53 mutations affect p53 lactylation or its functional consequences remains unknown (Zhang et al., 2018). Because p53 contributes to DNA damage-induced cell-cycle arrest and apoptosis, p53 lactylation could potentially weaken these responses and thereby reduce the sensitivity of GBM cells to radiotherapy. However, this proposed relationship has not been directly demonstrated and should be examined in GBM models with defined TP53 status.

#### 3.3.2 Enhancement of DNA repair and treatment resistance

Non-histone lactylation can enhance DNA repair in GBM cells, helping them escape therapy-induced death. In ALDH1A3-high GSCs, lactate accumulation induces XRCC1-K247la, strengthens its interaction with importin-α, and promotes its nuclear translocation. These changes enhance single-strand break repair and support cell survival after radiotherapy and TMZ exposure (Li G. et al., 2024). HAT1-catalyzed RPA1-K88la increases the binding of RPA1 to single-stranded DNA and promotes homologous recombination. Disrupting this pathway with a competitive peptide increases radiosensitivity in preclinical GBM models (Zhao Y. et al., 2026).

Non-histone lactylation can also activate resistance programs beyond canonical DNA repair. Inhibiting the TNF–CELSR2–p65–HDAC1-K200la–EPAS1 axis suppresses the mesenchymal-like transcriptional state and sensitizes GBM cells to TMZ. This finding indicates that site-specific lactylation can connect myeloid-derived signals with tumor cell state transitions and drug resistance (Gao et al., 2026).

Macrophage-derived lactate provides another link between DNA repair, stemness, and immune escape. Tumor-associated macrophages export lactate through MCT4, while GSCs take it up through MCT1. This process induces KU70-K317la, enhances KU70-dependent non-homologous end joining, reduces cytosolic DNA, and limits cyclic GMP–AMP synthase–stimulator of interferon genes (cGAS–STING) activation. Conversely, blocking lactate transport or KU70-K317la improves the response to immune checkpoint blockade in immunocompetent intracranial GBM models (Li et al., 2026). Representative histone and non-histone lactylation mechanisms reported in GBM and broader glioma models are summarized in Figure 1. Together, these findings suggest that lactylation may support resistance to genotoxic therapy through complementary mechanisms: XRCC1-K247la, RPA1-K88la, and KU70-K317la enhance different DNA repair pathways, whereas p53 lactylation may weaken DNA damage-induced cell-cycle arrest and apoptosis. Whether these mechanisms cooperate to promote radiotherapy resistance in GBM remains unknown and should be investigated in models with defined TP53 status.

**FIGURE 1:**
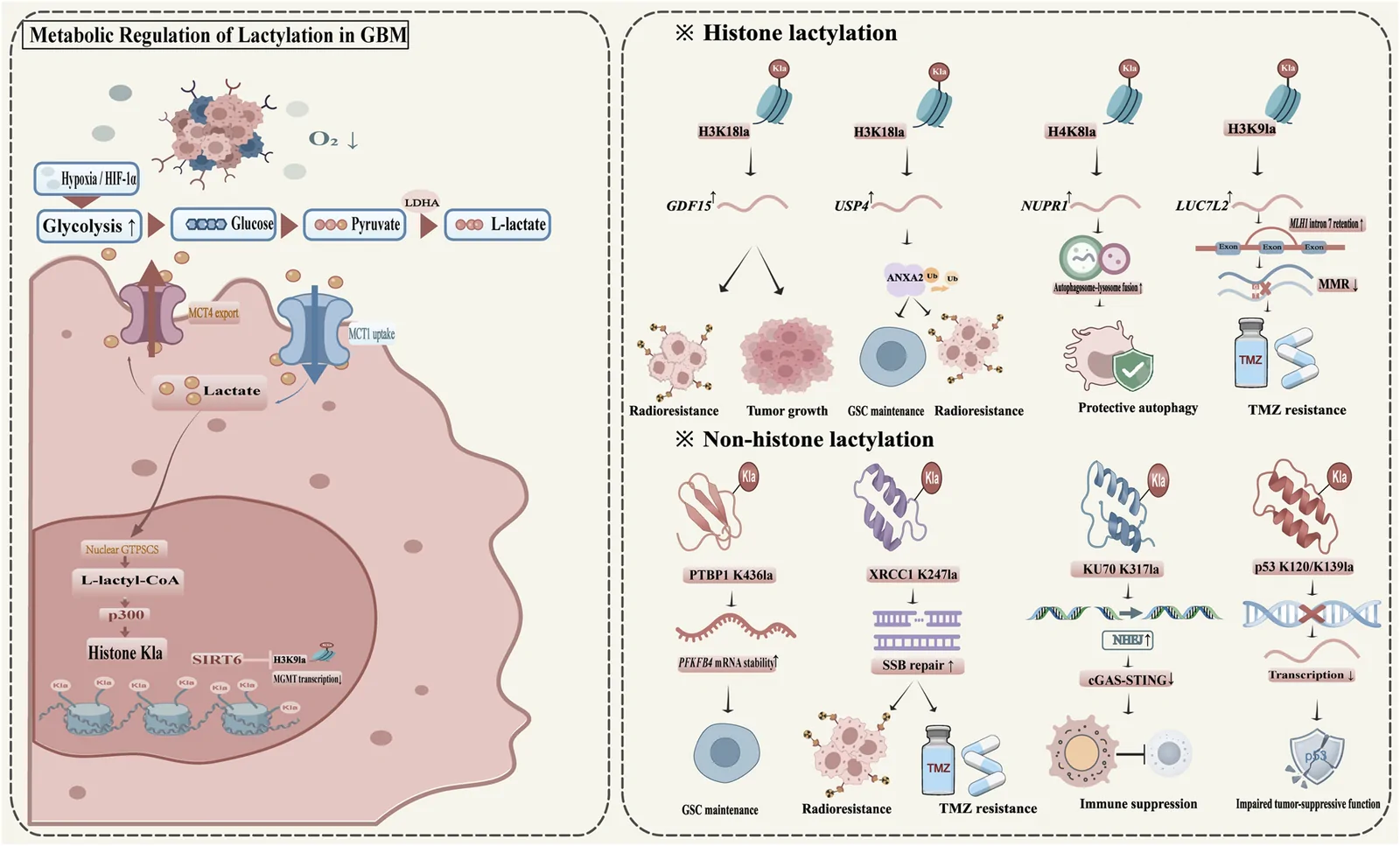
Metabolic regulation and functional consequences of histone and non-histone lactylation in glioblastoma.

#### 3.3.3 Potential links to ferroptosis and cuproptosis

Lactylation has recently drawn attention as a potential regulator of ferroptosis and cuproptosis. Evidence from other solid tumors suggests that non-histone lactylation may promote ferroptosis resistance by stabilizing ferroptosis-related proteins or antioxidant molecules, increasing glutathione synthesis, and limiting lipid peroxidation. These mechanisms, however, have not yet been directly validated in GBM. Given the extensive metabolic reprogramming of GBM and its intrinsic resistance to ferroptosis-inducing therapies, an important question is whether lactylation helps tumor cells evade ferroptosis or cuproptosis. It also remains unclear whether this process offers a targetable metabolic vulnerability.

### 3.4 Heterogeneity and microenvironmental effects of lactylation in GBM

GBM is highly heterogeneous at the molecular, cellular, and spatial levels. It contains distinct transcriptional subtypes and plastic malignant cell states, each linked to particular metabolic and microenvironmental features (Wang et al., 2017; Neftel et al., 2019; Garofano et al., 2021). Its spatial organization also produces regional differences in oxygen availability, glycolytic activity, and cellular composition within individual tumors (Ravi et al., 2022). Lactate accumulation and lactylation are therefore likely to differ across cell populations and tumor regions (Guyon et al., 2022; Wang S. et al., 2024). These variations are further shaped by metabolic interactions among tumor cells, infiltrating immune cells, and surrounding brain tissue (Wang S. et al., 2024; Hagihara et al., 2021). Lactylation in GBM is thus better understood as a dynamic, spatially organized process influenced by malignant cell states, local metabolism, and the surrounding microenvironment.

#### 3.4.1 Potential differences in lactylation among molecular subtypes

GBM comprises classical, proneural, mesenchymal, and other molecular subtypes (Wang et al., 2017). Each has distinct metabolic and microenvironmental features and may consequently show a different lactylation profile (Garofano et al., 2021). The mesenchymal subtype is often linked to hypoxia, increased glycolysis, and inflammation. It also expresses higher levels of *LDHA* than the other major subtypes. Together, these features suggest a stronger metabolic basis for elevated lactylation (Neftel et al., 2019; Garofano et al., 2021).

Direct quantitative comparisons of global and site-specific lactylation across GBM subtypes are still lacking, however, and the profiles of other subtypes remain poorly defined. Subtype identities are also not fixed. Malignant cells can shift between cellular states in response to microenvironmental conditions and treatment pressure. Lactylation differences may therefore reflect dynamic cell states rather than stable subtype classifications (Gao et al., 2026; Neftel et al., 2019).

#### 3.4.2 Spatial heterogeneity of cellular states and lactylation

Even within the same GBM tumor, lactylation levels may differ among malignant cells. GSCs generally show more active glycolysis and higher histone lactylation than differentiated tumor cells (Wang S. et al., 2024). Spatial context adds another layer of variation. Hypoxic regions favor glycolysis and lactate production, whereas better-oxygenated tumor margins may take up and use lactate more efficiently (Guyon et al., 2022; Brat et al., 2004). Lactylation may therefore vary with both cellular state and location (Wang S. et al., 2024; Neftel et al., 2019; Ravi et al., 2022). However, spatial transcriptomics usually captures the expression of genes linked to lactate metabolism and lactylation rather than protein lactylation itself (Ravi et al., 2022). These expression patterns should therefore not be treated as direct evidence of protein lactylation. Confirming such patterns will require spatial proteomics, modification-specific antibodies, or site-resolved mass spectrometry.

#### 3.4.3 The lactate–lactylation axis and the GBM immune microenvironment

The immune effects of lactylation in GBM are highly cell-type dependent. Tumor cells, microglia, and monocyte-derived macrophages can produce or take up lactate, thereby activating distinct lactylation programs across the tumor microenvironment (Wang S. et al., 2024; De Leo et al., 2024). These findings highlight the importance of myeloid-cell metabolism, impaired phagocytosis, and intercellular lactate exchange beyond a solely T-cell-centered framework. Although H4K8la-mediated *FAP* transcription has been linked to stromal remodeling and immune suppression in other cancers, its relevance to GBM remains unconfirmed.

Lactate can also acidify the TME, serve as a metabolic substrate, and activate receptors independently of lactylation (Brown and Ganapathy, 2020; Li et al., 2022; Longhitano et al., 2022). Future studies should therefore distinguish direct lactate effects from lactylation-mediated immune regulation in GBM.

#### 3.4.4 Differences in lactylation among tumor, peritumoral, and healthy brain tissues

Lactate metabolism and protein lactylation are part of normal brain physiology and are not inherently pathological (Hagihara et al., 2021). In GBM, however, glycolysis and lactate accumulation are more pronounced (Guyon et al., 2022). Animal studies have shown higher nuclear lactylation in GBM cells than in surrounding normal brain cells. Histone lactylation is also higher in GSCs than in normal neural stem cells (Wang S. et al., 2024).

Peritumoral brain tissue, however, is neither healthy brain nor tumor tissue. In GBM, this region forms a complex environment shaped by lactate accumulation, inflammatory mediators, hypoxia, cerebral edema, and infiltrating tumor cells. Peritumoral lactylation has not been directly measured, so it cannot be assumed to be intermediate between tumor and healthy brain. However, the high cellular and metabolic heterogeneity of peritumoral areas suggests that lactylation may vary across cell types and spatial niches (Ravi et al., 2022). Direct comparative studies of lactylation across tumor, peritumoral, and healthy brain tissues remain lacking. Such comparisons are needed to separate tumor-specific lactylation from physiological or reactive changes in the surrounding brain.

## 4 Therapeutic strategies targeting the lactate–lactylation axis in GBM

Lactylation is increasingly recognized as a central regulator of cancer progression and a potential therapeutic target. Current interventions mainly aim to inhibit lactate production or transport, regulate lactylation through key enzymes, or block downstream resistance pathways. This section summarizes progress in lactylation-targeted therapy in solid tumors, including GBM, and examines the feasibility of combining these emerging approaches with radiotherapy, chemotherapy, immunotherapy, and molecularly targeted agents. Future directions in this field are also discussed. Representative preclinical strategies targeting lactate metabolism and lactylation-associated mechanisms in GBM and broader glioma models are summarized in Table 2.

**TABLE 2:** Representative preclinical interventions targeting lactate metabolism or lactylation-associated mechanisms in glioblastoma and broader glioma models.

Category	Tested intervention	Lactylation-related mechanism	Therapeutic effect	References
Lactate metabolism	Stiripentol (LDH inhibition)	LDH activity↓ → lactate metabolism/metabolic symbiosis↓	GBM growth↓	Guyon et al. (2022)
	MCT1 inhibition	MCT1-mediated monocarboxylate transport↓	GSC viability and proliferation↓	Takada et al. (2016)
Histone lactylation	GTPSCS knockdown	Nuclear lactyl-CoA↓ → H3K18la at the *GDF15* promoter↓	Glioma growth↓; radiosensitivity↑	Liu et al. (2025)
TMZ combination	Gemfibrozil or GW7647 + TMZ	PPARα–*ACOX1*↑ → PKM2/glycolysis/lactate↓ → H3K18la↓	TMZ sensitivity↑	Wang et al. (2025a)
	MDL-800 + TMZ	SIRT6-mediated H3K9 delactylation↑→ H3K9la enrichment at the *MGMT* promoter↓→ *MGMT* transcription↓	TMZ sensitivity↑	Zou et al. (2025)
	TREM1-inhibitory peptide + TMZ	TREM1-dependent MES-MDM signaling↓ → HDAC1-K200la/*EPAS1* axis↓	MES-like transition↓; TMZ sensitivity↑	Gao et al. (2026)
Immunotherapy	Oxamate + CAR-T cells	Lactate/H3K18la↓ → H3K18la enrichment at the *ENTPD1* (CD39), *NT5E* (CD73), and *CCR8* promoters↓ → gene expression↓	CAR-T-cell activity↑	Sun et al. (2023b)
	DCA + anti-CD47 antibody	Lactate↓ → CBX3–p300-dependent histone Kla↓ → *CD47* transcription↓	Phagocytosis and antitumor activity↑	Wang et al. (2024b)
	Macrophage MCT1 or TNFSF9 knockdown	Lactate uptake↓ → macrophage H3K18la-dependent *TNFSF9* transcription↓	M2-like polarization and glioma growth↓	Li et al. (2024c)
	MCT4/MCT1 inhibition or KU70-K317la targeting + ICB	TAM→GSC lactate transfer↓ → KU70-K317la/NHEJ↓ → cytosolic DNA/cGAS–STING↑	Antitumor immunity↑; additive therapeutic benefit with ICB	Li et al. (2026)
Radiotherapy	*USP4* knockdown + RT	H3K18la-dependent *USP4* transcription↓ → ANXA2 deubiquitination and stability↓ → BMX–STAT3 signaling↓	GSC maintenance↓; Radiosensitivity↑	Tu et al. (2025)
	RPA1-K88la competitive peptide + RT	RPA1-K88la↓ → RPA1–ssDNA binding and homologous recombination↓	Radiosensitivity↑	Zhao et al. (2026b)
Chemoradiotherapy	D34-919 + TMZ/RT	ALDH1A3–PKM2 interaction and PKM2 tetramerization↓ → lactate production↓ → XRCC1-K247la and nuclear translocation↓ → SSB repair↓	Chemoradiotherapy sensitivity↑	Li et al. (2024b)
Antiangiogenic therapy	BMAL1 silencing or K123R mutation + bevacizumab	H3K18la at the *ARNTL* (BMAL1) promoter↓ and BMAL1-K123la-dependent nuclear translocation↓ → *VEGFA* transcription↓	Bevacizumab sensitivity↑	Wang et al. (2026)

### 4.1 Direct targeting of lactate metabolism and lactylation

Targeting lactylation has emerged as a potential anticancer strategy that links metabolic regulation with epigenetic control (Lv et al., 2025; Rho and Hay, 2025). Current approaches act at three main levels. At the upstream metabolic level, lactate production or transport can be inhibited to limit substrate availability. In GBM, the lactate dehydrogenase (LDH) inhibitor stiripentol reduces lactate production and suppresses tumor growth and invasion (Guyon et al., 2022). MCT1 blockade also decreases GSC survival and proliferation (Takada et al., 2016). However, neither approach specifically targets lactylation. LDH and MCT inhibition also affects energy metabolism, redox balance, extracellular acidification, and lactate signaling. Their antitumor effects therefore cannot be attributed entirely to reduced lactylation without site-resolved measurements or rescue experiments.

Another strategy is to modulate lactylation by targeting its writer or eraser enzymes. HAT1, p300, GTPSCS, ACSS2, and several HDAC and sirtuin family members have been implicated in this process (Wang S. et al., 2024; Liu et al., 2025; Moreno-Yruela et al., 2022; Zhao Y. et al., 2026; Zhu et al., 2025). In GBM, however, most evidence comes from genetic perturbation or competitive peptide studies. Well-characterized small-molecule modulators and systematic *in vivo* safety data remain scarce. These enzymes are also not specific to lactylation, as they regulate other acylations, transcriptional programs, and metabolic processes. Site-specific detection, mutagenesis, and rescue experiments are therefore essential for linking an observed phenotype to lactylation.

The most direct approach is to block lactylation at a defined protein site. In colorectal cancer, Pep4 inhibits XLF-K288la, impairs DNA repair, and improves the efficacy of 5-fluorouracil (Jin et al., 2025). In GBM, a competitive peptide targeting RPA1-K88la suppresses homologous recombination and sensitizes tumor cells to radiotherapy (Zhao Y. et al., 2026). Yet therapeutic peptides still face practical limitations, including short half-lives, enzymatic degradation, poor cellular uptake, and restricted passage across the blood–brain and blood–tumor barriers. Overall, interventions targeting lactate supply, regulatory enzymes, or functional substrates offer different advantages, but enzyme- and site-directed strategies still require rigorous validation in GBM.

### 4.2 Combination with chemotherapy

Chemotherapy is a cornerstone of systemic cancer treatment, and combining lactylation inhibition with chemotherapy has shown substantial therapeutic potential across cancer types. Studies in other cancers have linked histone and non-histone lactylation to chemoresistance through ferroptosis suppression, enhanced DNA repair, and increased drug efflux, and have suggested that targeting specific lactylation events may improve chemotherapy efficacy (Sun X. et al., 2023; Zhang K. et al., 2025; Sun C. et al., 2025; Li X. et al., 2025). In GBM, lactylation is an experimentally supported contributor to TMZ resistance (Yue et al., 2024; Li G. et al., 2024). As discussed above, histone and non-histone lactylation can reduce TMZ sensitivity by enhancing DNA repair, impairing mismatch repair, and promoting resistance-associated transcriptional states (Yue et al., 2024; Li G. et al., 2024; Gao et al., 2026). Therapeutically, PPARα activation reduces lactate production and histone lactylation, whereas MDL-800 promotes SIRT6-mediated H3K9 delactylation. A TREM1 inhibitory peptide suppresses the HDAC1-K200la-associated mesenchymal program, and all three interventions enhance TMZ sensitivity in preclinical GBM models (Wang Z. C. et al., 2025; Zou et al., 2025; Gao et al., 2026).

### 4.3 Combination with immunotherapy

Although immunotherapy has transformed the treatment of several cancers, acquired resistance remains a major challenge. One important contributor is the immunosuppressive TME created by lactate accumulation and lactylation (Wang S. et al., 2024; De Leo et al., 2024; Li et al., 2026). In preclinical GBM xenograft models, oxamate-mediated inhibition of lactate production enhanced chimeric antigen receptor T-cell (CAR-T-cell) activity. Oxamate treatment reduced H3K18la and CD39 and CD73 expression while suppressing CCR8 expression and adenosine metabolism (Sun T. et al., 2023).

Direct evidence from GBM now links intercellular lactate transport to innate immune suppression. Based on the KU70-K317la mechanism described above, blocking lactate transfer from TAMs to GSCs or targeting KU70-K317la enhances antitumor immunity and improves the response to immune checkpoint blockade in immunocompetent GBM models (Li et al., 2026). These findings support a potential combination strategy. However, its clinical efficacy and the safety of disrupting lactate exchange in the brain remain unknown.

Similar approaches have been tested in other tumors. In gastric cancer, β-alanine inhibits the lactylation writer AARS1, reduces protein lactylation, limits extracellular matrix remodeling and CD8^+^ T-cell exclusion, and improves the response to anti-PD-1 therapy (Huang et al., 2026). Targeting lactylation may therefore help counter tumor-induced immunosuppression and improve immunotherapy.

### 4.4 Combination with molecularly targeted therapy

In GBM, H3K18la-mediated *ARNTL* transcription and BMAL1-K123la-dependent nuclear translocation provide direct preclinical evidence that lactylation promotes bevacizumab resistance by increasing *VEGFA* transcription (Wang et al., 2026). Epigenetic silencing of *RFX7* has also been proposed as part of a transcriptional axis linking lactate metabolism to the response to immune checkpoint therapy (Han et al., 2026). Together, these findings extend the therapeutic relevance of lactylation. However, prospective biomarker validation and pharmacologically tractable, site-selective interventions are still needed.

Outside GBM, histone and non-histone lactylation have also been linked to resistance to lenvatinib in hepatocellular carcinoma, niraparib in ovarian cancer, and enfortumab vedotin in bladder cancer through the H3K18la–*HECTD2*, H4K12la–MYC–*RAD23A*, and YTHDC1 lactylation pathways, respectively (Dong R. et al., 2025; Lu et al., 2025; Xing et al., 2025). In GBM, available evidence shows that lactylation can maintain stemness (Tu et al., 2025), enhance DNA repair (Li G. et al., 2024), support metabolic adaptation (Zhou et al., 2025), and suppress antitumor immunity (Li et al., 2026). Targeting lactylation could therefore weaken several resistance mechanisms and improve responses to agents targeting DNA repair, metabolism, or other molecular pathways. Nevertheless, direct evidence for these combinations in GBM remains limited, and most approaches are still at the mechanistic or preclinical stage.

## 5 Challenges and future directions

Lactylation is increasingly recognized as a metabolically responsive protein post-translational modification with important roles in cancer biology. However, many fundamental questions remain unresolved, particularly in highly heterogeneous tumors such as GBM. Future studies need to clarify how lactylation is regulated, how it operates across cell populations, how it changes over space and time, and how it can be measured reliably. Its clinical value must also be assessed with care.

### 5.1 Regulation of lactylation and modification crosstalk

A major challenge is that the molecular machinery controlling lactylation remains incompletely defined. Several enzymes have been proposed to act as writers or erasers, whereas only a small number of lactylation readers have been identified, including BRG1 (Hu et al., 2024), DPF2 (Zhai et al., 2024), and BRD9 (Wei et al., 2026). This gap matters because reader proteins are expected to decode lactylation signals and connect metabolic cues with transcriptional regulation, protein stability, and signal transduction. Without a clearly defined writer, reader, and eraser system, it is difficult to distinguish causal effects of lactylation from metabolic changes that merely occur alongside it. Systematic identification and functional validation of lactylation readers should therefore be a priority.

Lactylation also interacts with other epigenetic and post-translational modifications, including RNA modification, phosphorylation, ubiquitination, and SUMOylation (Lu et al., 2024; Tu et al., 2025; Wang Z. et al., 2025; Shan et al., 2025). These interactions suggest that lactylation may amplify its biological effects through interconnected modification networks rather than a single linear pathway. Given the metabolic plasticity and intratumoral heterogeneity of GBM, it will be especially important to determine the sequence, dependence, and functional consequences of these events.

### 5.2 Lactylation and the immune microenvironment

Beyond tumor-cell-intrinsic regulation, lactylation is emerging as an important determinant of the tumor immune microenvironment. Studies have shown that it can influence immune-cell differentiation, cytokine production, and immune checkpoint expression, thereby promoting immunosuppression (Hao et al., 2024; Llibre et al., 2025; Sun M. et al., 2025; Chen et al., 2026). In colorectal cancer, macrophage H3K18la suppresses RARγ expression and activates TRAF6–IL-6–STAT3 signaling, providing site- and pathway-level evidence that lactylation can reshape myeloid-cell function (Li X. et al., 2024). Because GBM cells rely heavily on glycolysis and continuously release lactate, lactylation-mediated immune regulation may contribute substantially to immune escape. Even so, the key pathways and dominant targets remain poorly defined. Addressing this gap will require systematic analysis of lactylation across distinct immune-cell populations in GBM.

Lactate is not confined to the cells that produce it. It can be exchanged through MCTs and reshape lactylation in recipient cells, effectively serving as a form of metabolic communication. In conventional glioma cell-line and co-culture models, hypoxia increased lactate production by glioma cells. This glioma-derived lactate was taken up by macrophages through MCT1, where it increased H3K18la-associated *TNFSF9* transcription and promoted M2-like polarization (Li M. et al., 2024). In regulatory T cells, MOESIN lactylation strengthens TGF-β signaling and enhances immunosuppression (Gu et al., 2022). In GBM, intercellular lactate-driven lactylation may help shape and sustain interactions between tumor cells and the surrounding microenvironment. Co-culture systems, patient-derived organoids, and *in vivo* metabolic tracing will be needed to define these processes more clearly.

### 5.3 Spatiotemporal dynamics and physiological functions of lactylation

The spatiotemporal behavior of lactylation during GBM evolution remains largely unknown. It is unclear whether lactylation programs are reconfigured during malignant progression, recurrence, or adaptation to radiotherapy, chemotherapy, and immunotherapy. Most available evidence comes from static analyses and therefore cannot capture changes over time or under treatment pressure (Yang et al., 2022; Liu et al., 2024; Tu et al., 2025; Zhou et al., 2025).

Spatial heterogeneity adds another layer of complexity. Hypoxic tumor cores and relatively oxygen-rich invasive margins occupy distinct metabolic states and may develop region-specific lactylation patterns with different biological consequences. Integrating spatial transcriptomics and proteomics with site-specific lactylation profiling will be essential for resolving these spatially encoded mechanisms (Han et al., 2025; Xu et al., 2025; Zhang et al., 2025a). The physiological roles of lactylation in the normal brain also need careful examination. It should not be assumed from the outset that lactylation is inherently oncogenic. A balanced understanding of its normal functions is necessary for evaluating the safety of future therapies and will depend on more precise, specific, and spatially resolved detection methods.

### 5.4 Detection standards and biomarker validation

Methodological limitations continue to restrict lactylation research. Mass spectrometry can resolve specific modification sites but may lack sensitivity for low-abundance events. Antibody-based assays, by contrast, can be affected by limited specificity and cross-reactivity (Wang et al., 2023). Emerging tools may help. These include the YnLac chemical probe for *in situ* lactylation labeling (Sun et al., 2022), hyperpolarized [1–^13^C]pyruvate magnetic resonance spectroscopy for metabolic imaging (Nelson et al., 2013), and machine-learning predictors such as FSL-Kla and Auto-Kla (Jiang et al., 2021; Lai and Gao, 2023). Nevertheless, standardized workflows, benchmarking strategies, and quality-control criteria remain essential for reproducibility and meaningful comparisons across studies.

The value of lactylation-related features for patient stratification in GBM is also at an early stage. Most current models are based on lactylation-related gene expression in public transcriptomic datasets such as The Cancer Genome Atlas (TCGA), the Chinese Glioma Genome Atlas (CGGA), and the Gene Expression Omnibus (GEO), rather than direct measurements of protein lactylation in patient tumors (Han et al., 2025; Xu et al., 2025). Although some studies include external validation or time-dependent receiver operating characteristic (ROC) analyses, their gene-selection methods, risk cutoffs, and patient populations vary considerably. Prospective multicenter validation and direct comparison with established clinical markers are still lacking. Predicted treatment responses are also largely computational rather than derived from actual treatment cohorts. For now, these models should be viewed as exploratory stratification tools rather than validated diagnostic, prognostic, or predictive biomarkers. Reliable clinical application will require direct measurement of protein lactylation and standardized biomarker validation.

### 5.5 Priorities and future directions for clinical translation

Translating lactate- or lactylation-targeted strategies into GBM treatment faces several barriers. These include inadequate drug delivery to the brain, potential metabolic toxicity in normal brain tissue, and limited target and patient selectivity (Hagihara et al., 2021; Narsinh et al., 2024). Metabolic differences across tumor regions and between patients further complicate dose selection and identification of suitable candidates. Current evidence therefore does not support the broad use of approaches that globally inhibit lactate metabolism or lactylation.

Future studies should prioritize targets with clearly defined modification sites, causal evidence, and confirmed *in vivo* functions. Their efficacy, safety, and brain distribution should then be tested in patient-derived organoids and immunocompetent orthotopic GBM models. Pharmacodynamic studies should confirm target engagement across spatially distinct tumor regions, particularly at the infiltrative edge and in residual tumor cells, while distinguishing lactylation-specific effects from the broader consequences of disrupting lactate metabolism. Patient stratification should combine direct lactylation measurements with metabolic and spatial information. Hyperpolarized pyruvate MRI has already been used to visualize lactate metabolism noninvasively in prostate cancer (Nelson et al., 2013). Importantly, however, it measures lactate metabolic flux rather than protein lactylation itself. Whether this technology can support patient selection, response monitoring, or lactylation research in GBM will require prospective clinical validation.

## 6 Conclusion

Current evidence supports lactylation as a regulatory interface linking metabolic reprogramming with epigenetic control, oncogenic signaling, and remodeling of the tumor immune microenvironment across multiple cancers, including GBM. By dynamically modifying histone and non-histone proteins, lactylation can promote tumor cell plasticity, stemness, DNA repair, immune escape, and therapeutic resistance.

Nevertheless, the evidence is uneven. Several site-specific mechanisms have been validated in preclinical GBM systems, whereas other conclusions derive from conventional glioma cell lines, broader glioma models, lactate interventions without direct lactylation measurements, or expression-based bioinformatic analyses. The spatiotemporal dynamics, cell-type specificity, and physiological functions of lactylation in the brain remain incompletely defined.

Future work should move beyond global inhibition toward interventions directed at defined cellular contexts, modification sites, and metabolic states. Integrating spatial and single-cell approaches with site-resolved lactylomics, standardized validation, biomarker-guided stratification, and brain-appropriate delivery will be essential for determining whether lactylation can be translated into a clinically useful biomarker or therapeutic vulnerability in GBM.
